# Machine Learning for Subtyping Concussion Using a Clustering Approach

**DOI:** 10.3389/fnhum.2021.716643

**Published:** 2021-09-30

**Authors:** Cirelle K. Rosenblatt, Alexandra Harriss, Aliya-Nur Babul, Samuel A. Rosenblatt

**Affiliations:** ^1^Advance Concussion Clinic Inc., Vancouver, BC, Canada; ^2^Division of Sport & Exercise Medicine, Department of Family Practice, Faculty of Medicine, University of British Columbia, Vancouver, BC, Canada; ^3^Department of Astronomy, Columbia University, New York, NY, United States

**Keywords:** concussion, artificial intelligence, cluster analysis, interdisciplinary, rehabilitation, mild traumatic brain injury, complexity

## Abstract

**Background:** Concussion subtypes are typically organized into commonly affected symptom areas or a combination of affected systems, an approach that may be flawed by bias in conceptualization or the inherent limitations of interdisciplinary expertise.

**Objective:** The purpose of this study was to determine whether a bottom-up, unsupervised, machine learning approach, could more accurately support concussion subtyping.

**Methods:** Initial patient intake data as well as objective outcome measures including, the Patient-Reported Outcomes Measurement Information System (PROMIS), Dizziness Handicap Inventory (DHI), Pain Catastrophizing Scale (PCS), and Immediate Post-Concussion Assessment and Cognitive Testing Tool (ImPACT) were retrospectively extracted from the Advance Concussion Clinic's database. A correlation matrix and principal component analysis (PCA) were used to reduce the dimensionality of the dataset. Sklearn's agglomerative clustering algorithm was then applied, and the optimal number of clusters within the patient database were generated. Between-group comparisons among the formed clusters were performed using a Mann-Whitney U test.

**Results:** Two hundred seventy-five patients within the clinics database were analyzed. Five distinct clusters emerged from the data when maximizing the Silhouette score (0.36) and minimizing the Davies-Bouldin score (0.83). Concussion subtypes derived demonstrated clinically distinct profiles, with statistically significant differences (*p* < 0.05) between all five clusters.

**Conclusion:** This machine learning approach enabled the identification and characterization of five distinct concussion subtypes, which were best understood according to levels of complexity, ranging from Extremely Complex to Minimally Complex. Understanding concussion in terms of Complexity with the utilization of artificial intelligence, could provide a more accurate concussion classification or subtype approach; one that better reflects the true heterogeneity and complex system disruptions associated with mild traumatic brain injury.

## Introduction

Mild traumatic brain injury (mTBI) is a growing public healthcare concern, and presents a substantial burden to patients, families and health care systems (Langer et al., 2020). The incidence rate of this silent epidemic has significantly increased over the past decade, and accounts for the majority of all reported traumatic brain injury cases (Rao et al., 2017). While the majority of patients recover within 3 months, up to 30% of patients experience persistent concussion symptoms, affecting their ability to return to school, work, and activities of daily living (Dennis et al., 2019; Permenter et al., 2021). Given the significant increase in concussion and economic burden to health care systems, there is a need for effective and efficient evaluation of these injuries by healthcare professionals to implement accurate and timely management strategies.

Concussion reflects in a variety of affected systems and areas of co-occurring disruption that requires interdisciplinary management, an approach that is universally recommended in consensus statements and best-practice clinical guidelines (Collins et al., 2016; McCrory et al., 2017; Schneider et al., 2019). It is the heterogeneous nature of concussion, from its etiology and pathophysiology, to its individual clinical presentation and variable recovery trajectories, that has made its management especially challenging for the clinician or primary care physician. Moreover, in regions or health care systems where interdisciplinary care falls outside insured or core medical services, translating concussion best-practices in the application of the interdisciplinary treatment model represents a further barrier toward effective concussion management and meaningful progress in addressing the concussion epidemic.

Growing evidence of the complexity of concussion has given rise to the development of clinical subtypes, with steady empirical support (Collins et al., 2014; Ellis et al., 2016, 2018; Kontos and Collins, 2018; Lumba-Brown et al., 2020), and is considered a valuable tool toward informing clinical decision making, treatment planning and the conceptualization of targeted rehabilitation pathways. Similar to Collins et al. (2014) early delineation of six distinct sport-related concussion subtypes based on patient-reported symptoms following one-week post-injury, subsequent subtypes, or post-concussion disorders, have been organized according to symptoms, impairments or a combination of affected systems.

However, these subtypes may be vulnerable to bias in their conceptualization (Langdon et al., 2020), or inherently limited by the application of discipline specific expertise. Certainly, there is a disproportionate body of research on sport related concussion with notable gaps in our understanding of mTBI associated with motor vehicle accidents and other causes. Moreover, the complexity of concussion and its multi-factorial nature does not align with the approaches that assimilate the dimensionality of concussion into its basic units of impairment. A reductionism approach–that isolates a single factor, or combination of factors, and assumes them to be the cause of injury or impairment (Hulme and Finch, 2015)–may be useful in understanding causal, linear relationships (Bittencourt et al., 2016), however the non-linear, multifaceted entity that is concussion may require more sophisticated methods to capture the complex determinants that influence outcomes. As Langer et al. (2020) suggested, such a model requires “testing in an overview of empirical evidence focusing on data driven clustering of symptoms” into concussion subtypes. Kontos and Collins (2018), identified the need for mapping symptom clusters across various domains that quantified symptom clusters of concussion into objective deficits in functional outcome domain measures.

Analyses that integrate Artificial Intelligence (AI) and embrace a systems approach to concussion, with inherent non-linearity and complex, dynamic interactions, may improve our ability to identify patterns of system disruption in such multidimensional injuries as concussion. Recent AI work has used machine learning to predict symptom resolution following sport-related concussion (Bergeron et al., 2019). While another study used a clustering approach on vestibular and balance diagnostic data, and demonstrated two clinically distinct groups, patients with prominent vestibular disorders and others with no clear vestibular or balance impairment (Visscher et al., 2019). Specifically in consideration of the heterogeneity of concussion and noting the ways in which this complicates research efforts, Kenzie et al. (2018) utilized causal loop diagramming to visualize relationships between concussion injury factors, including pathophysiology, deficits, symptom persistence and recovery trajectories.

Advances in machine learning can provide a distinct practical advantage to healthcare providers (Davenport and Kalakota, 2019). The advent of using machine learning in addressing complex healthcare questions is already underway, demonstrating promise in automating and assisting in clinical diagnoses and treatment response (Garcia-Vidal et al., 2019; Nakata, 2019; Stevens et al., 2019). Compared to traditional statistics, machine learning can identify non-linear relationships and high-order interactions between multiple variables, where traditional statistics fall short (Bergeron et al., 2019). Thus, machine learning could more appropriately address multifaceted and complicated human health conditions, such as concussion.

Hierarchical agglomerative clustering, is a unsupervised, bottom-up, machine learning approach that can identify subgroups from complex data and provide an opportunity to classify clinical patterns as well as create novel representations of clinical profiles (Hassan et al., 2020). There are substantial implications for research on concussion subtypes, but also the utilization of machine learning to help in interpreting overall assessment results and summarizing multiple parameters to identify which features or combination of features discriminate between clinical profiles. This approach aligns with the heterogeneity, complexity, and diversity of concussion.

The purpose of this study was to determine whether a bottom-up, unsupervised, machine learning approach, could provide insight into different concussion clinical profiles by using objective outcome measures, including the Patient-Reported Outcomes Measurement Information System (PROMIS), Dizziness Handicap Inventory (DHI), Pain Catastrophizing Scale (PCS), and Immediate Post-Concussion Assessment and Cognitive Testing Tool (ImPACT). Utilization of self-administered, objective outcome measures, without the costly barriers of interdisciplinary concussion assessment were prioritized for this study.

## Materials and Methods

### Participants

In this retrospective study design, a cluster analysis was used on 275 patients in the initial intake database of the Advance Concussion Clinic, in Vancouver, British Columbia, Canada. Patients who were 18 years of age and older as well as completed the PROMIS (Version 2.1), DHI, PCS and ImPACT between January 2018 to December 2019 were included in the analysis. Patients were excluded from the analysis if, >5% of the patient's data were missing from the database, the patient did not allow the use of their data for research purposes, the patient did not complete the objective outcome measures at their initial assessment.

### Database

The Advance Concussion Clinic initial intake database consists of patient reported outcome measures obtained from PROMIS, DHI, PCS, and ImPACT. PROMIS is set of person-centered measures that evaluates and monitors domains of pain interference, fatigue, depression, anxiety, sleep disturbance, cognitive concerns and abilities, physical function and social function on a one to five numeric rating scale, as well as an average pain intensity score, on a zero to 10 numeric rating scale (Cella et al., 2010). The DHI is a 25-item form that evaluates a patients self-perceived handicapping effects imposed by vestibular dysfunction (Jacobson and Newman, 1990). The PCS is a 13-item scale that assesses three aspects of catastrophizing: helplessness, rumination and magnification (Sullivan et al., 1995). ImPACT is a computerized neurocognitive testing measure, which consists of six cognitive test modules. These six modules are utilized to generate four composite scores: verbal memory, visual memory, visual-motor processing speed, and reaction time. A number of studies have reported on the test's validity and utility in identifying subtle cognitive changes associated with concussion (Schatz et al., 2006; Van Kampen et al., 2006; Broglio et al., 2007; Alsalaheen et al., 2016). The Acute Concussion Evaluation (Gioia et al., 2008) and Concussion Grading Scale (CGS) were also extracted from the initial assessment. The CGS is a 21-item self-report measure that records symptom severity using a 7-point Linkert scale. Studies demonstrate that the CGS scale is able to discriminate between concussed and non-concussed patients (Schatz et al., 2006; Broglio et al., 2007).

### Data Analysis

Data extracted from the Acute Concussion Evaluation were generated using descriptive statistics in SPSS statistical software (IBM Corp, 2017) and are reported as mean and standard deviation. For the cluster analysis, participant data were imported into Python (Python, Wilmington, DE: Python Software Foundation) and analyses were carried out using the scikit-learn toolkit (Abraham et al., 2014). Patient data were stored into a single matrix, where each row represented one patient and each column one variable. As a first step to reduce multicollinearity, a correlation matrix was used and redundant features were discarded. Moderate correlation areas (*r* > 0.55) were reduced to the element with the highest inter-patient variability (Schober et al., 2018). A principal component analysis (PCA) was then applied to reduce the dimensionality of the dataset (Jolliffe and Cadima, 2016). A PCA extracts information needed to explain the highest amount of variance within the dataset and in turn produces a set of new orthogonal variables called principal components (Feeny et al., 2020). Following, sklearn's agglomerative clustering algorithm was used on the determined principal components.

Agglomerative clustering is a hierarchical bottom up clustering approach which groups objects into clusters based on n their similarity. It is particularly suited for datasets where clusters maybe unevenly shaped, of unequal size and unequally distributed across parameter space (Hirano et al., 2004). In this hierarchical cluster analysis, the model is initialized by assuming that each datapoint is an individual cluster (Feeny et al., 2020; Hassan et al., 2020). Similarity and linkage are the two parameters of greatest importance for agglomerative clustering. Similarity was calculated using the euclidean distance between two data-points in PCA space while linkage was measured as the variance of each cluster (ward linkage).

Ward linkage was chosen to minimize the variance of each cluster ensuring that assessment scores of patients in each cluster were maximally uniform (Hirano et al., 2004). For the remaining parameters we used the default sklearn. At each iteration of the algorithm, the clusters with the shortest distance merge. The distance between clusters containing multiple data values is calculated using the minimum distance between a point in cluster *x* and a point in cluster *y*. The algorithm will continue merging clusters until stopped or there is only one large cluster. Since agglomerative clustering begins by assigning each datapoint a cluster, very few assumptions are made about the data. This is one of the strengths of agglomerative clustering, compared to other clustering methods, such as k-means (Hassan et al., 2020).

**Figure 1:**
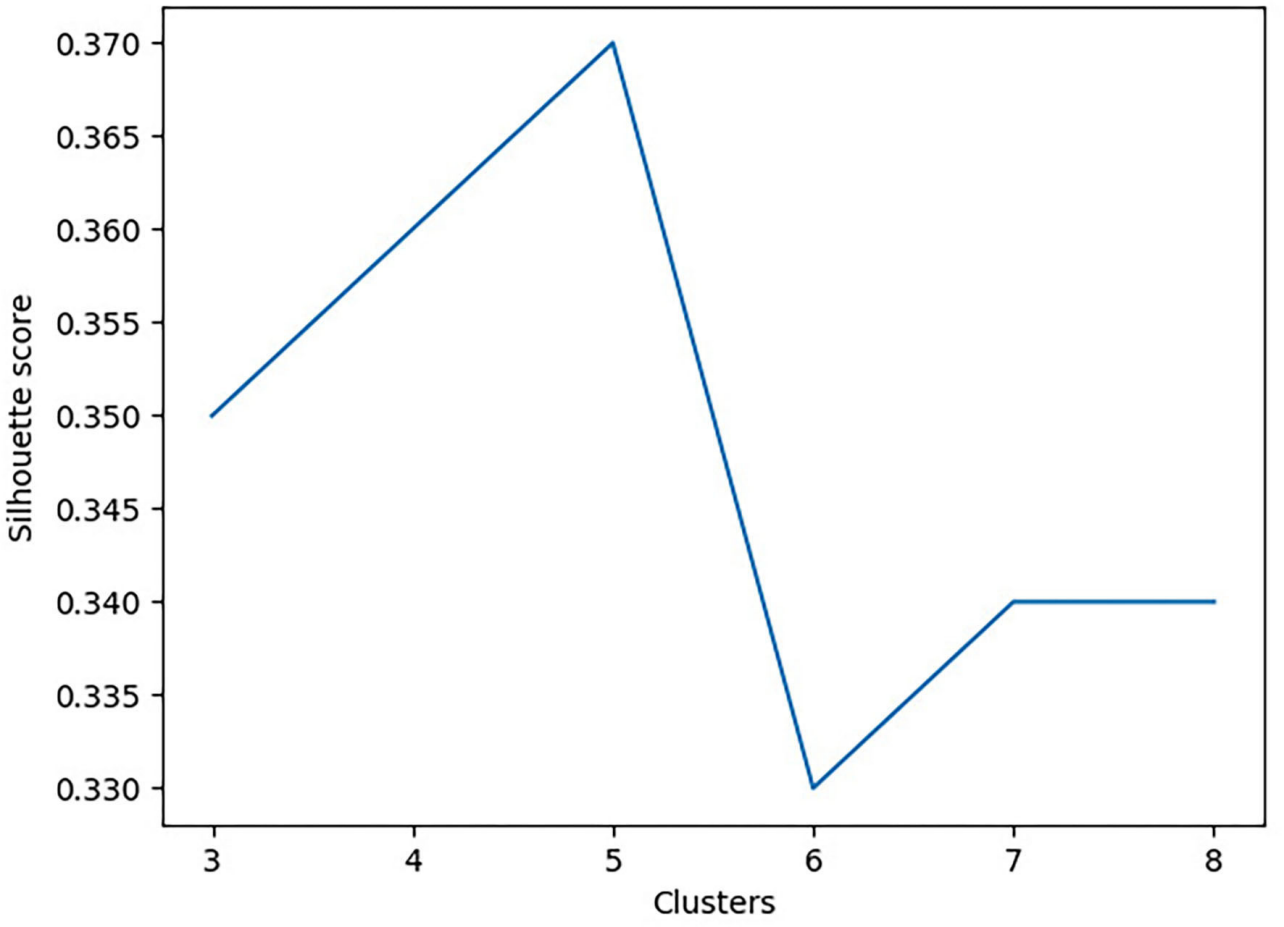
The Silhouette score as a function of the number of clusters.

To determine the optimal number of clusters, the Silhouette and Davies-Bouldin scores were used (Feeny et al., 2020; Hassan et al., 2020). The Silhouette score is used to determine the separation distance between the resulting clusters (i.e., measures how similar an object is to its own cluster, compared to other clusters). In contrast, the Davies-Bouldin score is a measure of similarity between each cluster. In this analysis, we aimed to minimize the Davies-Bouldin score to ensure each cluster is maximally different from the other clusters and maximize the Silhouette score to maintain maximal uniformity between values within a given cluster. Figure 1 shows the Silhouette score as a function of the number of clusters. To improve the stability of the cluster outcomes, the agglomerative clustering model started with 100 patients to capture the initial cluster properties. Following, an additional 100 patients were added to ensure that both the properties and profiles of each cluster did not change. Finally, all remaining participants were added to ensure cluster stability in that profiles and properties remained unchanged.

A Mann Whitney U test was used to determine significant differences between each assessment in each cluster against the same assessment in another cluster (i.e., pain interference in cluster one against pain interference in cluster two). A Bonferroni Correction was used on multiple comparisons (Ranstam, 2016). For all analyses, a statistical significance was assessed using *p* < 0.05 and confidence interval of 95% (Table 1).

**Table 1:** Means and standards deviations of the five determined clusters.

**Cluster**	**Outcome measures**													
	**PROMIS**									**DHI**		**ImPACT**		
	**Pain interference percentile**	**Pain intensity**	**Physical function and mobility percentile**	**Anxiety percentile**	**Depression percentile**	**Sleep disturbance percentile**	**Ability to participate in social roles percentile**	**Cognitive function percentile**	**Fatigue percentile**	**DHI total**	**DHI functional**	**Visual motor speed composite**	**Reaction time composite score**	**Memory composite (verbal) score**
2	50.97 (9.24)	0.22 (0.19)	50.80 (6.96)	48.6 (8.05)	46.04 (8.06)	43.69 (7.88)	51.63 (8.80)	32.86 (6.46)	48.27 (9.64)	5.91 (7.51)	2.52 (3.28)	37.54 (7.68)	0.69 (0.10)	89.83 (9.33)
3	60.23 (7.61)	0.43 (0.21)	42.82 (7.09)	60.22 (8.87)	55.71 (7.56)	54.27 (7.11)	39.95 (7.98)	43.39 (7.33)	61.20 (7.45)	22.70 (12.56)	9.63 (5.77)	38.62 (7.77)	0.67 (0.13)	90.20 (6.92)
1	61.42 (5.99)	0.46 (0.20)	40.82 (7.01)	57.71 (6.89)	55.17 (7.99)	55.01 (6.97)	40.03 (7.52)	42.86 (5.38)	59.94 (5.92)	32.77 (14.05)	12.85 (6.36)	28.37 (6.90)	0.86 (0.18)	62.45 (10.68)
0	67.76 (5.80)	0.62 (0.18)	36.35 (5.23)	66.65 (7.81)	62.23 (8.30)	58.48 (7.96)	34.12 (5.92)	49.57 (6.49)	68.24 (6.35)	60.94 (11.58)	24.97 (5.09)	30.50 (7.13)	0.80 (0.17)	79.63 (9.77)
4	69.94 (8.99)	0.73 (0.24)	33.98 (4.22)	65.88 (6.88)	63.46 (5.43)	66.87 (8.28)	30.41 (4.45)	51.69 (7.43)	69.46 (6.02)	71.75 (9.66)	30.13 (3.69)	20.53 (6.61)	1.10 (0.24)	47.19 (10.72)

*Mann Whitney U tests determined statistically significant in-between differences across all assessments in each of the clusters (p < 0.05)*.

## Results

### Participant Characteristics

The mean age of participants was 37.84 (SD = 12.50 years of age). Females represented 51% (*n* = 139) of the study population, males 48% (*n* = 133), and 1% (*n* = 3) did not disclose this information. The majority of concussions occurred following a motor vehicle accident (*n* = 148, 54%), and remaining mechanisms of injury were sport (*n* = 70, 25%), falls (*n* = 22, 8%), and other (*n* = 35, 13%). Loss of consciousness (LOC) was reported in 73 participants (27%), with 202 (73%) denying or not reporting LOC. The mean total for the CGS was 52.8 ± 29.8. Time since injury and litigation weren't specifically calculated.

### Outcome of Clustering Procedure

**Figure 2:**
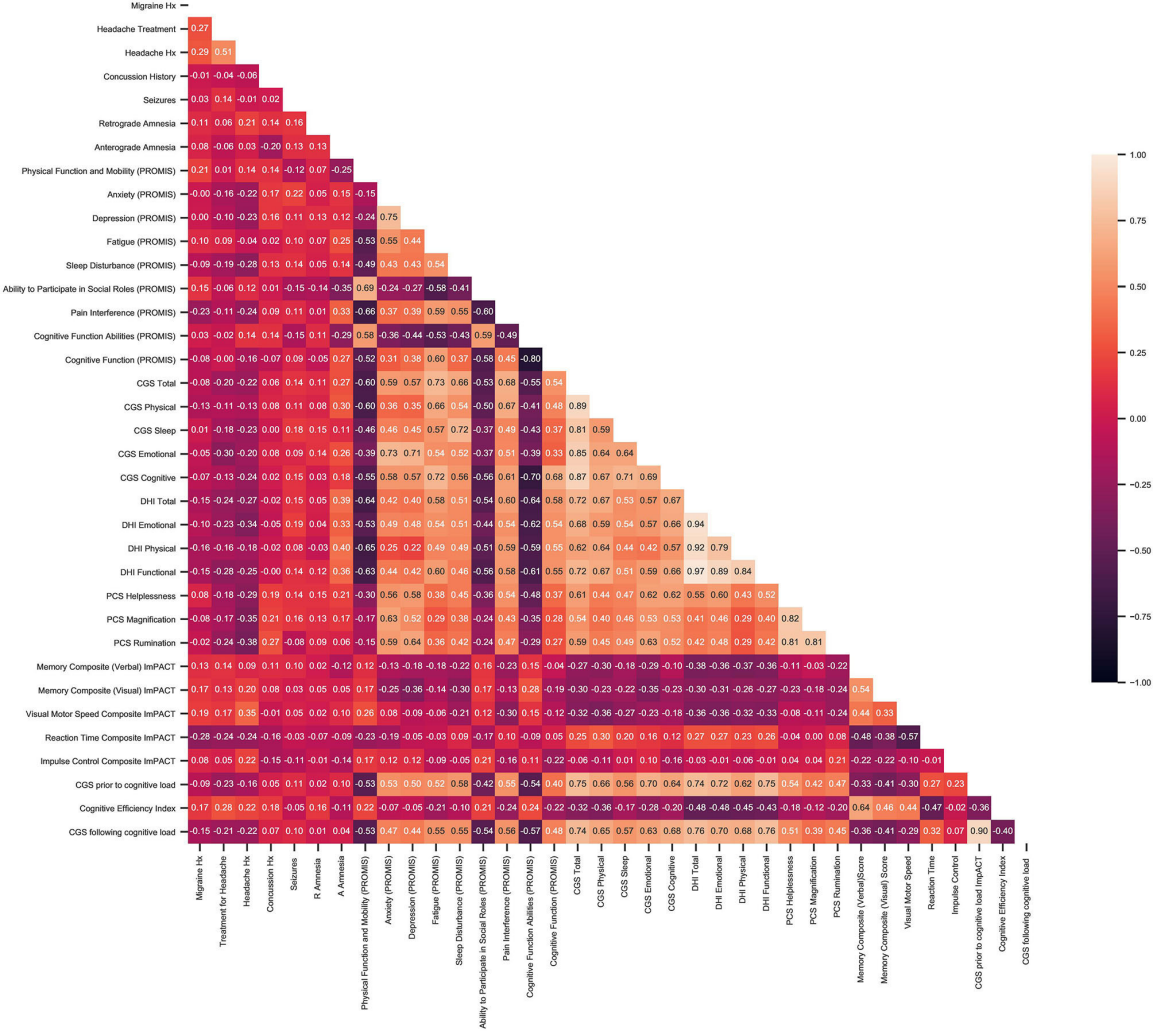
Results of correlation matrix between patient baseline characteristics and objective outcome measures including, PROMIS, CGS DHI, PCS, and ImPACT.

The correlation matrix extracted redundant features (*r* > 0.55), that were subsequently removed from the analysis (Figure 2). PROMIS parameters retained by the correlation matrix were Pain Interference, Fatigue, Depression, Anxiety, Sleep Disturbance, Physical Function and Mobility, Ability to Participate in Social Roles, and Cognitive Function as well as Pain Intensity on a numeric score. From DHI, the total score as well as the DHI Functional subgroup were retained. Lastly, ImPACT scores for reaction time, visual motor processing speed, and verbal memory were also used.

**Figure 3:**
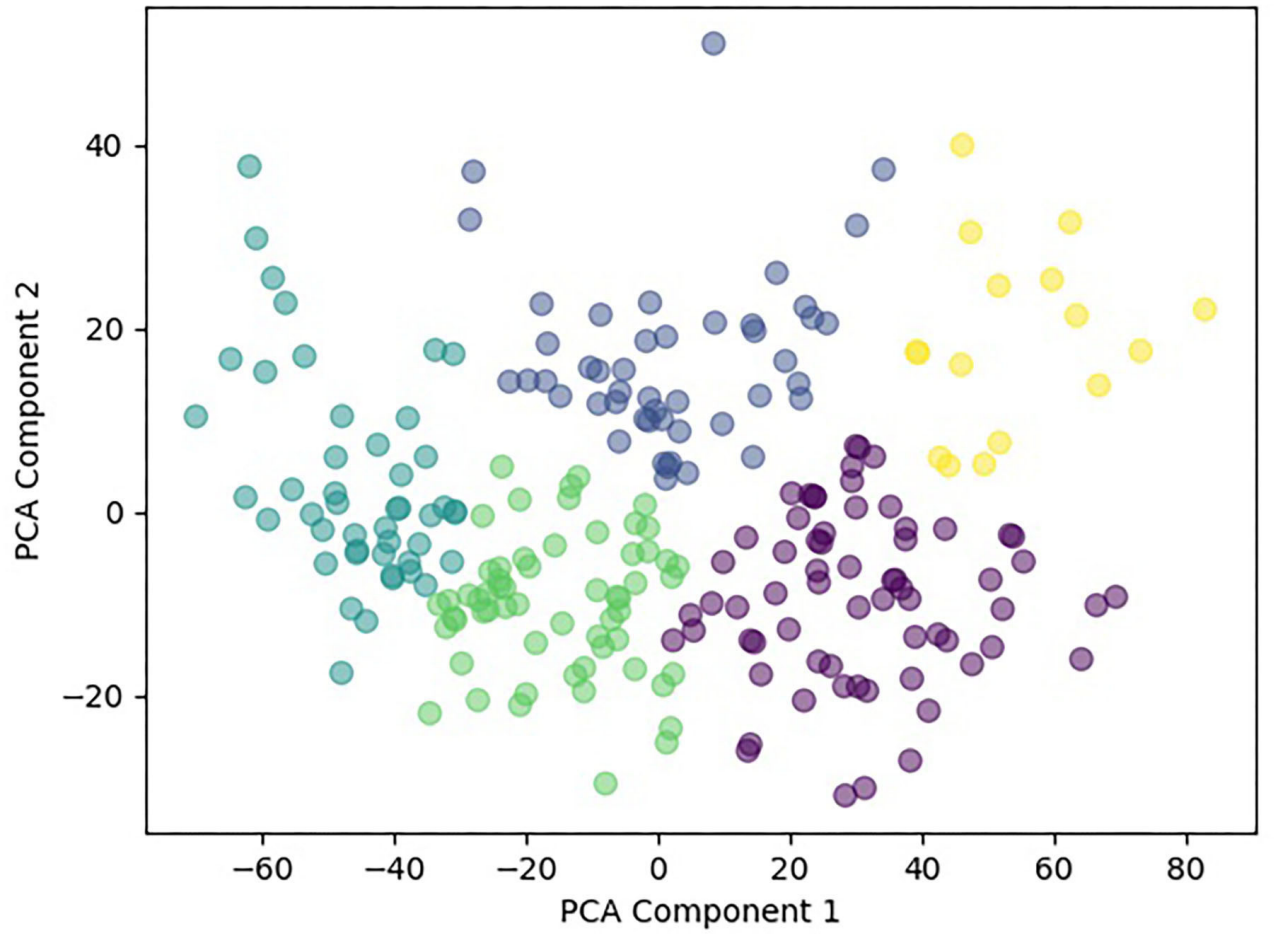
Scatterplot of the formed five clusters.

Principal component analysis further reduced the dimensionality of the dataset from 14 features to two features. Using sklearn's agglomerative clustering algorithm on the two principal components, the maximum silhouette score was 0.36 and minimum Davies Bouldin score was 0.83. Therefore, it was determined that the optimal number of clusters was five (Figure 3). Since all patients in the study presented with a concussion, the range of values for any given assessment was not very large. In addition, all patients suffered from pain and movement related symptoms so it was expected the Davies Bouldin score would lie closer to 1, indicating similarities between clusters. shows the clinically distinct five clusters. Mann Whitney U tests determined statistically significant in-between differences across all assessments in each of the clusters (*p* < 0.05).

## Discussion

Concussion is only seeming to grow in complexity as research and evidence advance our understanding of its heterogenous nature. From its etiology and pathophysiology, to its individual clinical presentation and variable recovery trajectories, clinical management has been especially challenging for the clinician or primary care physician. Research has varied widely in how it has dealt with this increasing complexity, with some seeming to lean into the complexity by highlighting systems approaches and recursive modeling (Schneider et al., 2019), while others argue that its heterogeneity is associated with mutually reinforcing biopsychosocial symptoms rather than a single entity (Iverson, 2019).

Clinically, management of the concussion patient has continued to grow in complexity as well, with increasing challenge for the primary care doctor or individual clinician, particularly in context of westernized medical frameworks and reimbursement systems. Multiple risk factors have been identified for prolonged recovery from concussion such as previous concussion history, developmental delay, headache history and psychiatric history, all of which require consideration together with a multitude of other variables that can influence outcomes. Clinical assessment of the concussion patient requires consideration of the above as well as potential interactions between them, all of which form the unique patterns of presentation in the individual concussion patient. AI is an ideal, and perhaps even necessary, partner, that can best support our ability to manage the complexity of concussion, toward gaining a better understanding of patterns that may improve our ability to diagnose and treat mTBI.

This study evaluated the use of an unsupervised, bottom-up, machine learning approach to analyze the clinical profiles of patients attending a private concussion clinic. Utilization of objective outcome measures provided a unique opportunity to engage machine learning with various concussion and non-concussion specific evidence-based metrics. Findings revealed five statistically significant and distinct clusters, each with unique patterns of system disruption.

Across each of these five clusters, 14 features were retained, which combined outcomes from PROMIS, DHI, and ImPACT. These 14 features were: Pain Interference, Pain Intensity, Physical Function and Mobility, Anxiety, Depression, Sleep Disturbance, Ability to Participate in Social Roles, Cognitive Function, Fatigue, DHI Total, DHI Functional, Visual Motor Speed, Reaction Time, and Verbal Memory. Reliable and validated self-administered outcome measures were specifically utilized to ensure the accessibility and affordability of this approach.

**Figure 4:**
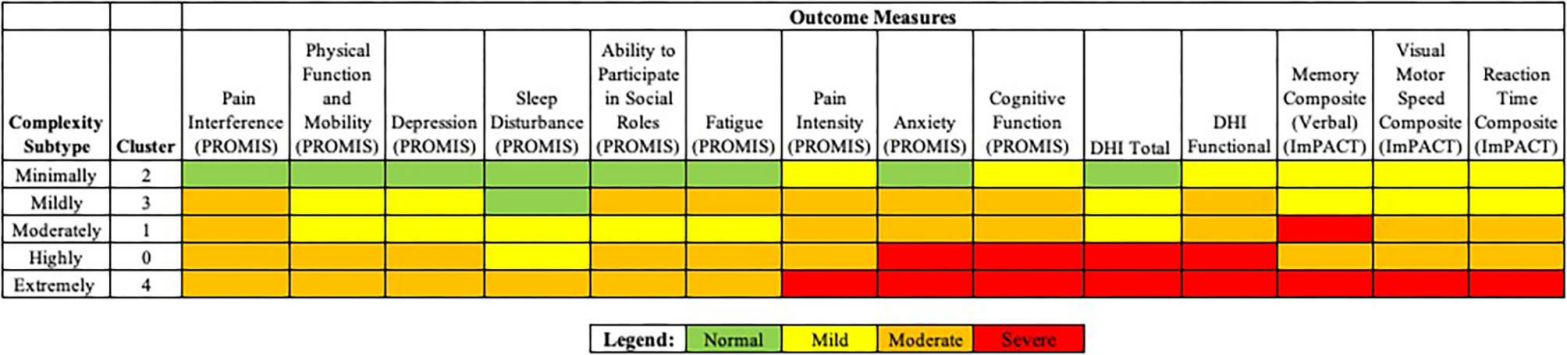
Concussion subtypes (clusters) according to complexity.

The authors considered each of five clusters, or concussion subtypes to be best understood in terms of Complexity, with the following classification system suggested for each concussion subtype: Minimally Complex (Cluster 2), Mildly Complex (Cluster 3), Moderately Complex (Cluster 1), Highly Complex (Cluster 0), Extremely Complex (Cluster 4) (Figure 4). Level of complexity was ranked according to number and relative severity of arenas affected. “Complexity” was considered to better align with the nature and particular features of concussive injury, informing specific and treatable features that support clinical decision making. The language of “complexity” was furthermore preferred to “severity” to promote perceived control, in both practitioner and patient, while avoiding the catastrophizing that might arise with a severity approach.

Historically, concussion classification approaches have grouped subtypes according to particular symptoms or a combination of affected systems. From the concussion profiles suggested by Collins et al. (2014) to the most recent subtypes as proposed by Lumba-Brown et al. (2020) and Kontos et al. (2020), the approach to subtyping has been grounded in heuristics derived from clinical experience and patient presentation or reporting.

The utilization of AI offered an opportunity to engage machine learning pattern recognition to explore what clinical concussion data can inform regarding concussion profiles. Indeed, this analysis revealed five unique and distinct subtypes with various *patterns* of system disruption, across multiple symptoms areas. This machine learning approach may be most appropriate for capturing the complexity and heterogeneity of the dynamic injury that is concussion, and in doing so, maximizing the potential of these subtypes to support and improve clinical decision making in concussions.

Reliable and validated self-administered outcome measures were specifically utilized to ensure the accessibility and affordability of this approach, offering a data-driven method toward enhancing clinical judgement and decision making without requiring commonly utilized clinicaly administered measures. The utilization of self-administered objective measures is of notable value in countries and regions that are more dispersed with less access to the recommended interdisciplinary concussion team otherwise needed to assess or screen the range of clinical areas affected in concussion.

Moreover, the Coronavirus pandemic emphasized the need for virtual tools to support and inform concussion care when in-person options are not available. The self-administered nature of the tools utilized in this analysis, as well the interdisciplinary value of the information provided, may well reduce costs or make affordable a reliable screening for those in a fee-for-service environment, as well as for the health systems that don't include interdisciplinary care among their insured services.

### Limitations and Future Directions

This clustering approach was selected to maximize the value of clinically meaningful data points that were derived from a large concussion population data set comprised of evidence-based outcome measures. This novel representation of concussion subtypes may help to guide interdisciplinary management, though further study is needed to assess its value in estimating recovery timelines and response to specific treatments. Future work should focus on a evaluating a wider variety of clustering algorithms to determine if they reveal further insights into the data; however, the uniformity in the clusters generated by the agglomerative clustering algorithm provide a first insight into the interconnectedness of systems affected by concussion. Further study is needed to understand the clinical application of these profiles and to explore the utility and feasibility of these subtypes within a clinical setting.

As the subtypes derived were based only on patients specifically presenting with symptoms associated with concussion, certainty regarding its diagnostic value cannot be ascertained. Extension of this cluster analysis to include healthy controls would support the validation of the current clinical profiles and aid in a diagnostic context.

Notably, many concussion subtypes have been developed based upon sport related concussions (SRC), while this research offers evidence of concussion subtyping more broadly applicable to concussions associated with other causes that are non-sports related. While the current sample represents a majority associated with motor vehicle accidents, results may be more generalizable than research that has focused on SRC alone. Further research would be needed to confirm the generalizability of results between these, and perhaps other, distinct groups.

### Conclusion

Classification of concussion according to subtypes may become a useful, if not essential, tool to support clinical diagnosis and treatment planning and is useful to concussion practitioners in directing and coordinating care, and in evaluating progress toward recovery. It stands to reason that as our knowledge and understanding of the complexity surrounding concussion grows, a more comprehensive approach is warranted.

This study demonstrated the novel opportunity of using AI to gain insight into the complex clinical profiles of concussion. By systematically analyzing evidence-based metrics, five clusters emerged that were not only clinically distinct, but could be used to develop a novel view of concussion complexity that better approximates the true heterogeneity of the injury. In turn, this could help inform as well as support clinical decision making, and interdisciplinary involvement more readily. Given the interdisciplinary nature of concussion assessment, and the importance of the interdisciplinary teams' findings in treatment planning and providing Clearance—both to learn and to sport—AI's work in automating, if not its diagnosis, it's management, would be useful to most primary care physicians and other clinicians involved in its management.

Furthermore, healthcare providers without specific training in concussion, and in those parts of the world where this expertise may be largely unavailable would also benefit. With the right collaboration and balance, the integration of AI with concussion subtyping can optimize otherwise elusive or incomplete concussion recoveries.

## Data Availability Statement

The datasets presented in this article are not readily available because these data are clinical information obtained from a private healthcare database. Requests to access the datasets should be directed to crosenblatt@advanceconcussion.com.

## Ethics Statement

Ethical review and approval was not required for the study on human participants in accordance with the local legislation and institutional requirements. The patients/participants provided their written informed consent to participate in this study.

## Author Contributions

CKR conceived the investigation with contribution by SAR. CKR and ANB designed the study. CKR, ANB, and SAR prepared the data. ANB trained and tested the algorithm with contribution by CKR. CKR and ANB interpreted the results. AH contributed to interpretation. AH and CKR drafted the paper and then substantially revised the paper with contributions by ANB. All authors contributed to the article and approved the submitted version.

## Conflict of Interest

The authors declare the potential following conflicts of interest with respect to the research, authorship, and/or publication of this article: AH, ANB, and SAR are employed by Advance Concussion Clinic. CKR is the Founder and Clinical Director of Advance Concussion Clinic.

## Publisher's Note

All claims expressed in this article are solely those of the authors and do not necessarily represent those of their affiliated organizations, or those of the publisher, the editors and the reviewers. Any product that may be evaluated in this article, or claim that may be made by its manufacturer, is not guaranteed or endorsed by the publisher.
